# Itaconate as an immunometabolic hub in host–pathogen interactions: Mechanisms, resistance, and therapeutic opportunities

**DOI:** 10.1080/21505594.2026.2749933

**Published:** 2026-10-15

**Authors:** Wei Zuo, Xinhui Wang, Jiaxiang Sun, Weiqi Guo, Beibei Zhang, Xinyu Wang, Jiangang Hu, Jingjing Qi, Mingxing Tian, Lei Deng, Shaohui Wang, Yanqing Bao

**Affiliations:** Shanghai Veterinary Research Institute, Chinese Academy of Agricultural Sciences, Shanghai, China

**Keywords:** Itaconate, immunometabolism, innate immunity, host-pathogen interactions, antimicrobial defense

## Abstract

Produced by IRG1 in macrophages, itaconate has emerged as an immunometabolic hub at the host-pathogen interface. This review integrates itaconate’s roles in antimicrobial defense. We first examine how itaconate inhibits pathogen metabolism by targeting key enzymes including isocitrate lyase, fructose-bisphosphate aldolase, and inosine monophosphate dehydrogenase. Beyond direct inhibition, itaconate reprograms host immunity through SDH suppression, Nrf2 activation, ATF3 induction, and context-dependent NLRP3 regulation, and promotes lysosomal biogenesis via TFEB. Conversely, pathogens have evolved counter strategies including enzymatic degradation, biofilm formation, and immune microenvironment remodeling. We also summarize preclinical evidence supporting itaconate and derivatives’ therapeutic potential for infection-associated conditions. Finally, we appraise theoretical gaps and methodological challenges, offering guidance for future investigation in this rapidly advancing field.

## Introduction

In recent years, a growing body of evidence has demonstrated that metabolism is intimately linked to immune responses. Functional changes in immune cells are consistently accompanied by profound metabolic adaptations to meet cellular energy demands. Upon pathogen infection, macrophages become significantly activated, resulting in enhanced inflammatory response and reprogrammed nutrient metabolism [1]. This activation not only increases glucose uptake and enhances glycolysis, but also exhibits partial suppression of the tricarboxylic acid (TCA) cycle, which in turn leads to accumulation of intermediates such as citric acid and cis-aconitic acid [2]. Meanwhile, the immune-responsive gene 1 (*Irg1*) is also upregulated. By catalyzing the decarboxylation of cis-aconitic acid, *Irg1* synthesizes itaconate in mitochondria. Although itaconate plays no significant roles in cellular energy metabolism, it functions as an endogenous anti-infective metabolite that contributes to an effective immune response, such as growth inhibition of pathogens, enhancement of lysosome biogenesis, and modulation of inflammatory responses [3–5]. Conversely, pathogens have also evolved defense strategies to counteract or exploit itaconate, which includes enzymatic degradation, establishment of metabolic bypass and remodeling of immune microenvironment [6–8]. Thus, focusing on itaconate, the host and pathogens are engaged in a dynamic arms race. In this review, we summarize recent advances defining itaconate as an immunometabolic hub at the host–pathogen interface, highlighting its multifaceted roles in antimicrobial defense, immune cell metabolic reprogramming, and pathogen counteradaptation. The therapeutic roles of itaconate and its derivatives in infectious and infection-associated inflammatory diseases are also discussed.

## A second life for itaconate

Itaconate was first described in 1836 by Swiss pharmacist Samuel Baup as a product of citric acid distillation [9]. In 1840, German scientist Gustav Crasso reported its synthesis via decarboxylation of cis-aconitate [10]. Beyond the laboratory, itaconate soon attracted industrial interest. In addition to chemical synthesis, itaconate can also be produced through microbial biosynthetic pathways. As early as 1931, Kinoshita K. identified the filamentous fungus *Aspergillus itaconicus* as a natural producer of itaconate [11]. Subsequently, in 1939, C.T. Calam and colleagues reported enhanced itaconate yields using a strain of *Aspergillus terreus*, thereby establishing a foundational platform for biotechnological production of this metabolite [12]. Owing to its trifunctional structure – featuring an unsaturated double bond and two carboxyl groups – itaconate is an attractive precursor for the synthesis of high-value chemicals and materials, including polymers. Consequently, itaconate has been widely used in the industrial production of plastics and rubber.

With ongoing research, the role of itaconate has expanded beyond chemical industries, revealing a “second life” as a key modulator of mammalian immunity. In 1995, C.G. Lee and colleagues cloned *Irg1*, which was found to be highly upregulated in murine peritoneal macrophages following lipopolysaccharide (LPS) stimulation [13]. However, for nearly two decades following its discovery, the function of *Irg1* remained unclear, as it was primarily studied in the context of interferon-inducible genes rather than metabolism. This changed in 2011 when C.L. Strelko and colleagues identified itaconate as an endogenous mammalian metabolite induced by LPS or interferon-γ (IFN-γ), and detected in the lungs of mice infected with *Mycobacterium tuberculosis* (*M. tuberculosis*) [14]. This study established, for the first time, a direct link between *Irg1* induction and itaconate production. Two years later, A. Michelucci et al. elucidated the mammalian biosynthetic pathway of itaconate, confirming that the protein encoded by *Irg1* is the key enzyme for itaconate production [3]. But the specific biological functions of itaconate were still unclear.

A pivotal breakthrough came in 2016 when V. Lampropoulou et al. demonstrated that bone marrow-derived macrophages (BMDMs) from *Irg1*-knockout mice produced elevated levels of pro-inflammatory cytokines such as IL-1β and IL-18 upon LPS stimulation, unveiling the immunoregulatory function of itaconate [15]. Since then, itaconate has become a central focus in immunometabolism research (Figure 1). Accumulating evidence highlights its diverse immune activities–including anti-inflammatory, antioxidant, antitumor, and antimicrobial effects–underscoring its involvement with a wide range of diseases. Among these, the antimicrobial functions of itaconate have gained particular interest, driven by its emerging role as a key effector molecule at the host–pathogen interface. In the following sections, we will focus on the multifaceted mechanisms by which itaconate exerts its antimicrobial activity and the counter strategies evolved by pathogens to subvert it.

**Figure 1.**
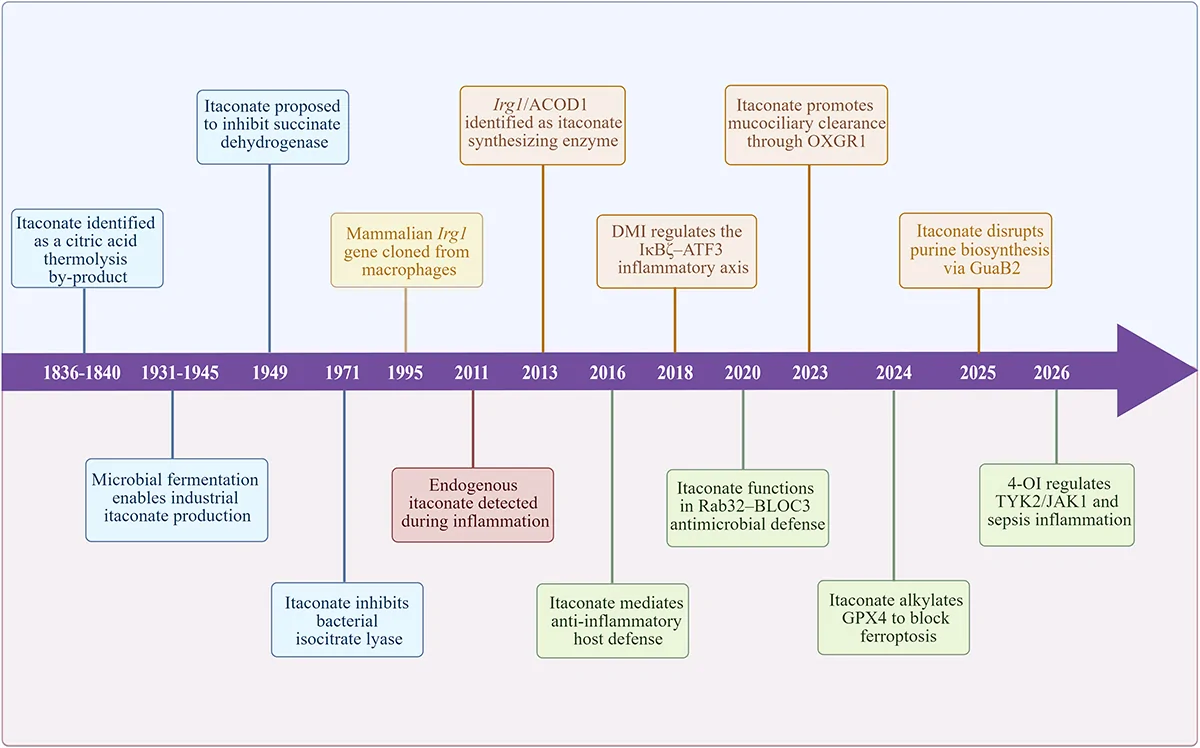
Timeline of major milestones in itaconate research. From its initial description as a product of citric acid distillation in 1836, itaconate research has evolved from chemical synthesis and industrial microbial production to antimicrobial metabolism, mammalian immunometabolism, and host–pathogen defense. Recent studies have established itaconate and its derivatives as multifaceted regulators of pathogen metabolism and host immune signaling. Abbreviations: ACOD1, aconitate decarboxylase 1; *Irg1*, immune-responsive gene 1; DMI, dimethyl itaconate; OXGR1, oxoglutarate receptor 1; BLOC3, biogenesis of lysosome-related organelles complex 3; GuaB2, inosine monophosphate dehydrogenase 2; 4-OI, 4-octyl itaconate; TYK2, tyrosine kinase 2; JAK1, Janus kinase 1; GPX4, glutathione peroxidase 4. Created with BioGDP.com.

## Mechanisms of itaconate in host defense: from direct restriction to immune-mediated protection

This section examines how itaconate contributes to host defense through three mechanistically distinct modes, which include direct pathogen restriction, immune-mediated pathogen clearance and immunomodulation and disease tolerance. Critically, the mechanisms discussed in this section have been elucidated using different molecular entities that are often collectively referred to as “itaconate.” These include itaconate (ITA), the free dicarboxylic acid produced intracellularly by macrophage ACOD1. When we refer to ITA, we specify whether it is endogenously produced (ACOD1-derived) or exogenously administered, as this distinction affects concentration, localization, and biological interpretation. Dimethyl itaconate (DMI), a cell-permeable methyl ester prodrug that is hydrolyzed by intracellular esterases to release ITA, but may achieve supraphysiological intracellular concentrations that engage targets not meaningfully modified by endogenous ITA. And 4-octyl itaconate (4-OI), a synthetic derivative in which an octyl chain substantially enhances membrane permeability and electrophilic reactivity. Since 4-OI alkylates a distinct set of signaling proteins that are not detectably modified by endogenously produced ITA atphysiological concentrations [16], it is therefore best regarded as a pharmacological lead compound with a distinct target landscape rather than ITA mimetic.

### Itaconate inhibition on bacterial growth

This section discusses the direct pathogen restriction of ITA-mediated host defenses. Endogenous, ACOD1-derived ITA targets and suppresses key metabolic enzymes in pathogens. In cell-free systems, exogenous added ITA has been used to identify and characterize these molecular targets. Additionally, endogenously produced ITA is selectively delivered into pathogen-containing vacuoles via specific transport pathways, enabling compartmentalized antibacterial action. Below, we systematically outline the principal metabolic targets of ITA in bacteria and the molecular mechanisms underlying its spatial delivery.

#### Itaconate inhibits the glyoxylate shunt

In most bacterial pathogens, glucose is catabolized via glycolysis to acetyl-CoA, which enters the TCA cycle–a central hub for generating biosynthetic intermediates and energy under nutrient-rich conditions [17,18]. While upon infection, intracellular pathogens often encounter glucose-limited conditions within the host, compelling them to utilize alternative carbon sources. A key adaptation is the activation of the glyoxylate cycle, which enables bacteria to generate biosynthetic precursors from C_2_ compounds such as acetate or fatty acids, allowing them to consume energy under glucose-limiting conditions [19,20].

The glyoxylate cycle involves two key enzymes: isocitrate lyase (ICL) and malate synthase. ITA primarily exerts antimicrobial effects by inhibiting the pathogen’s ICL and the glyoxylate shunt it depends on. This inhibition was first described in 1971, when researchers demonstrated that exogenous ITA (free acid) directly inhibited ICL from *Pseudomonas indigofera* in an activity assay conducted with purified enzyme. ITA showed potent inhibition of ICL, with activity detectable at 0.015 mM and an apparent inhibition constant (K′i) of approximately 0.007 mM, providing the first biochemical evidence that ITA targets the glyoxylate cycle through ICL inhibition [21]. Subsequent studies confirmed its physiological relevance, showing that ITA selectively inhibited *P. indigofera* growth by blocking ICL-dependent C_2_ assimilation. In ethanol-grown cultures, 10 mM ITA almost completely arrested growth, whereas 5 mM ITA mainly reduced growth rate, with minimal effects on glucose-grown cells [22]. The physiological relevance of this inhibition has been established in clinically important pathogens. For instance, *M. tuberculosis* upregulates ICL expression upon exposure to C_2_ carbon sources or after macrophage phagocytosis [23,24], and lack of ICL impairs *M. tuberculosis* survival in macrophages or mice [25]. Consistent with the essential role of this pathway, exogenous ITA inhibits *M. tuberculosis* growth under acetate-dependent conditions by targeting glyoxylate shunt metabolism. Specifically, ITA at concentrations of 25–50 mM completely inhibited *M. tuberculosis* growth when acetate was provided as the sole carbon source, whereas bacterial growth on non-glyoxylate shunt-dependent carbon sources was largely unaffected, highlighting ICL-dependent metabolism as a key metabolic vulnerability during pathogen adaptation [3]. This represents a mechanism of pathogen restriction in which ITA limits bacterial replication capacity through interference with a central metabolic enzyme. Similarly, in *Salmonella enterica* (*S. enterica*), ITA directly targets ICL through covalent modification of the catalytic cysteine residue Cys195. This itaconation event abolishes ICL activity, with ITA inhibiting wild-type ICL in a dose-dependent manner (IC_50_ = 2.6 mM), and disrupts glyoxylate shunt-dependent bacterial growth under carbon-limited conditions [26].

Beyond ICL, exogenous ITA (free acid) also inhibits methylisocitrate lyase (MCL), an enzyme in the 2-methylcitrate cycle that converts propionyl-CoA to succinate and pyruvate [27,28]. This pathway is particularly important for *M. tuberculosis* which metabolizes host-derived cholesterol, which generates propionyl-CoA, a toxic intermediate if allowed to accumulate [29]. In *M. tuberculosis*, ICL also exhibits MCL activity; therefore, inhibition of this dual-function enzyme by ITA disrupts propionyl-CoA detoxification through the methylcitrate cycle [30]. Under propionate-generating conditions, this combined inhibition enhances the suppression of *M. tuberculosis* growth through the accumulation of toxic metabolic intermediates (Figure 2). Thus, ITA contributes to antimicrobial defense by targeting key metabolic adaptations required for *M. tuberculosis* survival [3].

**Figure 2.**
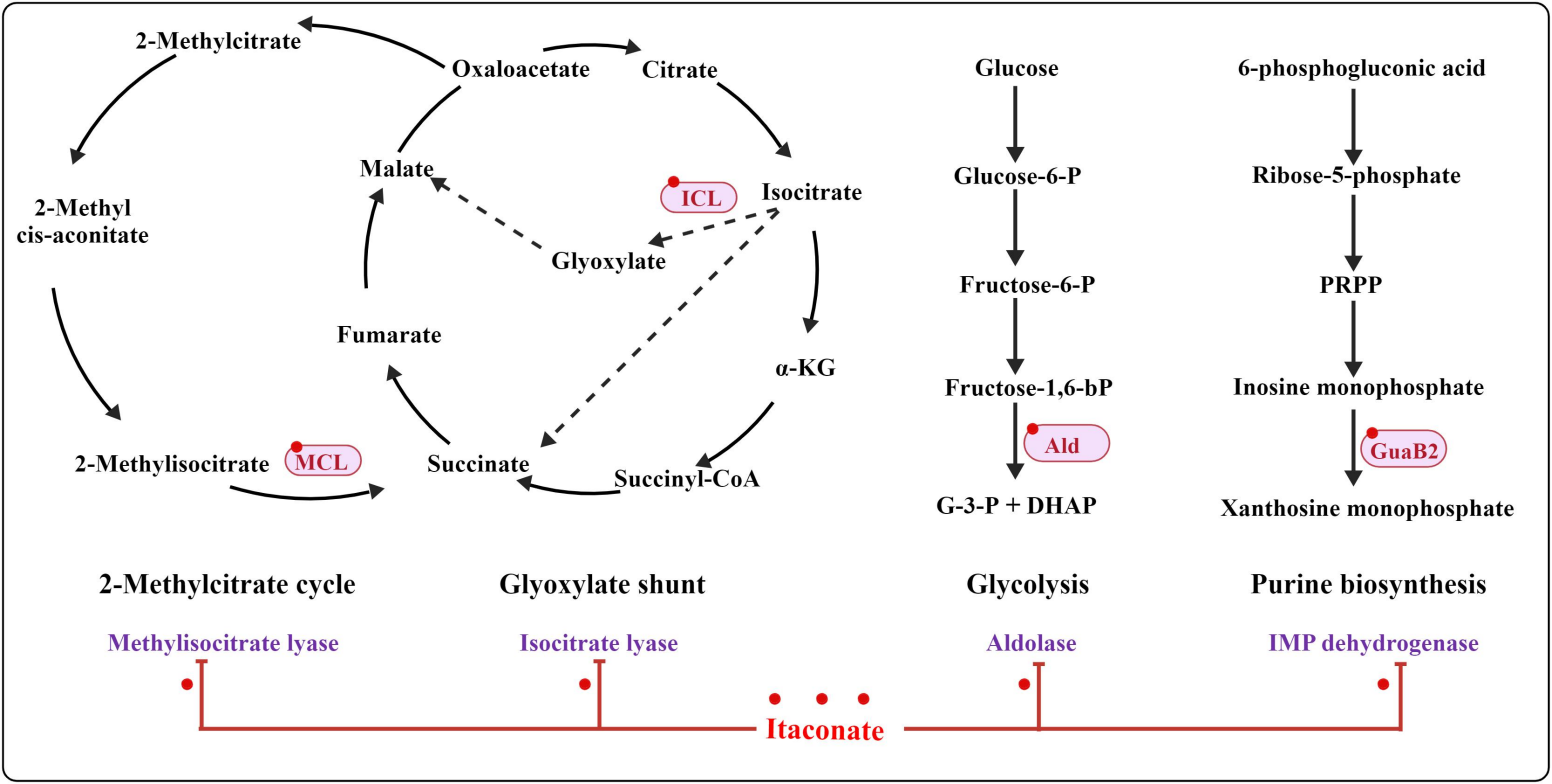
Antimicrobial activity: itaconate inhibition on pathogen metabolism. in the 2-methylcitrate cycle, itaconate suppresses methylisocitrate lyase (MCL), disrupting the conversion of 2-methylisocitrate to downstream intermediates. in the glyoxylate shunt, itaconate inhibits isocitrate lyase (ICL), blocking the cleavage of isocitrate to glyoxylate and succinate and thereby limiting carbon flux through this anaplerotic pathway. in glycolysis, itaconate targets fructose-1,6-bisphosphate aldolase (ald), preventing the formation of glyceraldehyde-3-phosphate (G-3-P) and dihydroxyacetone phosphate (DHAP) from fructose-1,6-bisphosphate. in purine biosynthesis, itaconate inhibits inosine monophosphate dehydrogenase 2 (GuaB2), restraining the conversion of inosine monophosphate (IMP) to xanthosine monophosphate (XMP). Created with BioGDP.com.

#### Itaconate inhibits glycolysis and purine biosynthesis

In addition to the glyoxylate shunt, recent studies have uncovered that ITA also targets other metabolic nodes. Using untargeted metabolomics and enzymatic assays, Priya et al. demonstrated that exposure of *M. tuberculosis* to 10–50 mM of ITA profoundly perturbs glycolysis and nucleotide metabolism in cell-free assays [6]. In particular, ITA directly bound to and inhibited fructose-bisphosphate aldolase (Ald) and inosine monophosphate dehydrogenase 2 (GuaB2) of *M. tuberculosis* in a concentration-dependent manner. At 10 mM ITA, ITA exhibited strong inhibitory effects on both enzymes, markedly reducing their enzymatic activities. Fluorescence quenching and docking analyses further confirmed specific, high-affinity interactions between ITA and the active-site residues of Ald (Asp95, His96, His212, His252, Gly253) and GuaB2 (Met85, Asp283, Asp374, Ala285, Ser339). These two enzymes catalyze essential steps in glycolysis and de novo purine biosynthesis, respectively. Ald mediating the cleavage of fructose-1,6-bisphosphate into triose phosphates, and GuaB2 catalyzing the conversion of IMP to XMP, a precursor for GMP synthesis. Inhibition of Ald by ITA leads to the accumulation of upstream intermediates such as glucose-6-phosphate and fructose-6-phosphate, coupled with depletion of dihydroxyacetone phosphate, reflecting a glycolytic bottleneck. Concurrently, inhibition of GuaB2 results in elevated intracellular levels of IMP and AICAR with concomitant reduction of XMP, indicating a blockade of the purine biosynthetic flux (Figure 2). Collectively, these inhibitory effects disrupt both energy metabolism and nucleotide synthesis, thus restricting *M. tuberculosis* growth in vitro and survival in macrophages or susceptible hosts.

#### Intracellular delivery of itaconate to pathogen-containing vacuoles

The direct antimicrobial effects of ITA require not only enzymatic target engagement but also appropriate subcellular localization. In the infected host cells, certain intracellular bacteria are enclosed within multilayered organelle-like structures which are called pathogen-containing vacuoles (PCVs). The exchange of materials and the maturation of these PCVs are tightly regulated by a range of host vacuolar proteins localized on their surfaces [31]. During *Salmonella* infection, Chen et al. demonstrated that the Rab32–BLOC3 pathway functions as a vesicular trafficking system that delivers endogenous ACOD1-derived ITA from mitochondria to *Salmonella*-containing vacuoles (SCV). Affinity purification coupled with LC–MS/MS followed by co-immunoprecipitation revealed an infection-enhanced interaction between Rab32 and *Irg1*, while live-cell imaging and three-dimensional structured illumination microscopy (3D-SIM) further showed that *Irg1*-positive mitochondria closely associate with SCV during infection. Using GFP- and luciferase-based *Salmonella* ITA biosensors, the authors demonstrated that Rab32–BLOC3 selectively enriches ITA within SCV, where the local concentration reaches approximately 6 mM, a bacteriostatic level sufficient to directly restrict intracellular *Salmonella* replication, without affecting total cellular ITA production. In contrast, genetic deletion of either Rab32 or the BLOC3 component HPS4 markedly reduced intravacuolar ITA accumulation while leaving overall cellular ITA levels unchanged, resulting in enhanced intracellular bacterial survival (Figure 3). Notably, *Salmonella typhimurium* (*S. typhimurium*) has evolved dedicated type III secretion effectors, SopD2 and GtgE, to neutralize this pathway, thereby preventing ITA delivery and restoring intracellular replication, underscoring strong evolutionary pressure to evade ITA-mediated killing [32]. Collectively, these findings establish ITA as a compartmentalized antimicrobial metabolite whose efficacy not only relies on its enzymatic targets but also on precise intracellular localization, highlighting spatial control as a critical determinant of metabolite-driven host defense.

**Figure 3.**
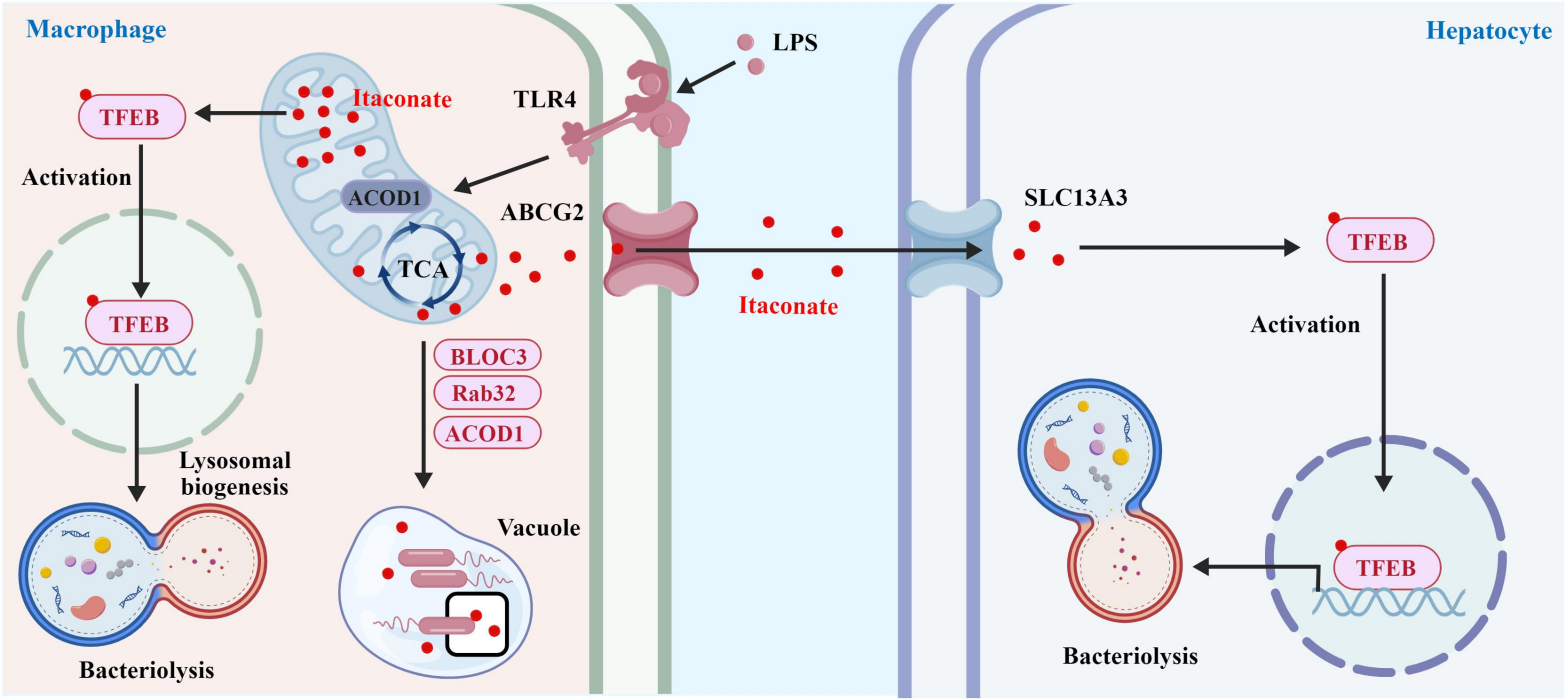
Spatial coordination of itaconate-mediated antibacterial defense in macrophages and hepatocytes. Upon LPS stimulation, TLR4 activation in macrophages induces ACOD1-dependent itaconate production within mitochondria. The Rab32-BLOC3 trafficking machinery directs itaconate to pathogen-containing vacuoles, mediating intravacuolar inhibition of bacteria. Concurrently, intracellular itaconate activates TFEB, driving lysosomal biogenesis and enhancing bactericidal capacity. ABCG2 mediates itaconate export, limiting intracellular accumulation. Extracellular itaconate can be imported into hepatocytes via SLC13A3, where it similarly activates TFEB and promotes lysosome-dependent bacteriolysis. Created with BioGDP.com.

### Itaconate-mediated pathogen clearance and immunomodulation/disease tolerance

Unlike the direct enzymatic inhibition, the mechanisms discussed here involve reprogramming of host immune cell activity to create an intracellular environment unfavorable for pathogen survival, as well as to restrain the excessive inflammatory responses that drive tissue damage. Both endogenously produced ITA and synthetic derivatives (DMI, 4-OI) are discussed, with the molecular entity explicitly identified in each subsection. We further specify whether each mechanism primarily achieves pathogen clearance or disease tolerance, such as suppression of immunopathology without necessarily reducing pathogen burden.

#### Itaconate contributes to intracellular bacteria killing by mediating lysosome synthesis

Recent studies have revealed that endogenously produced ITA also acts as an immune signaling molecule capable of activating the lysosomal defense system in macrophages. Upon pathogen recognition via TLR4, macrophages upregulate ACOD1 expression, leading to increased endogenous ITA production [33]. Intracellularly accumulated endogenous ITA alkylates cysteine 212 (C212) of the transcription factor TFEB, thereby inhibiting mTORC1-mediated phosphorylation at serine 211 (S211) and disrupting its interaction with 14–3-3 proteins. This modification promotes TFEB nuclear translocation. Once translocated into the nucleus, TFEB functions as a master regulator of lysosomal biogenesis, upregulating lysosome-related gene expression and consequently enhancing lysosome formation, maturation, and the capacity to eliminate intracellular pathogens [4]. This TFEB-driven lysosomal enhancement represents pathogen clearance, active elimination of microbes, distinct from the pathogen restriction mediated by direct enzyme inhibition mentioned above.

The magnitude of the ITA-driven lysosomal response is tightly controlled by metabolic transport mechanisms. The ATP-binding cassette transporter ABCG2 (localized at the plasma membrane) facilitates the efflux of ITA from macrophages, thereby limiting its intracellular accumulation and attenuating TFEB-dependent lysosomal biogenesis [4]. Importantly, the immunomodulatory effects of ITA are not restricted to immune cells. ITA released by activated macrophages can be taken up by nonimmune parenchymal cells, particularly hepatocytes, via the dicarboxylate transporter SLC13A3. This uptake similarly activates TFEB and induces lysosomal biogenesis, endowing these non-classical immune cells with bactericidal capabilities [34]. Such an intercellular metabolic relay enables systemic amplification of lysosome-dependent antimicrobial defense, extending the protective effects of ITA beyond the immune compartment (Figure 3).

#### Itaconate and derivatives reprogram immune cell metabolism to limit pathogen survival and tissue damage

Beyond its role as an antimicrobial metabolite, ITA has gained the attention of immunologists because of its immunomodulatory properties (Figure 4), which are still being actively explored. Below, we review ITA’s function that reprograms host immune cell metabolism and signaling pathways to establish an intracellular environment unfavorable for pathogen survival, enhances cellular bactericidal capacity, and mitigates tissue damage resulting from excessive immune activation. The latter function represents disease tolerance, which preserves host organ function without necessarily reducing pathogen burden. Where distinct, we specify whether the evidence derives from endogenously produced ITA, exogenously administered ITA (free acid), or pharmacological treatment with DMI or 4-OI, as these entities differ substantially in target engagement as mentioned above.

**Figure 4.**
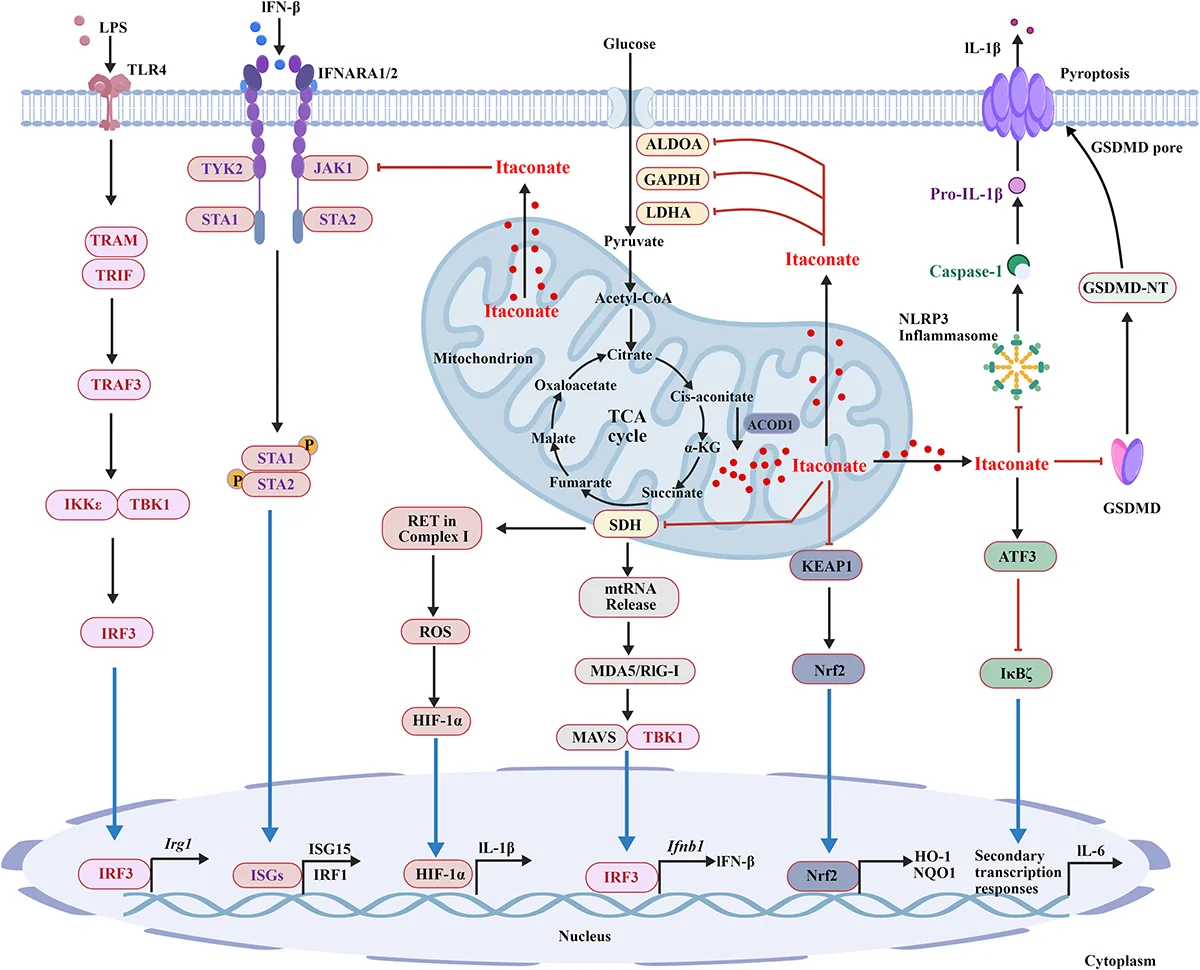
Itaconate-mediated immunometabolic reprogramming in macrophages. Upon LPS stimulation, TLR4 signaling activates the TRAM/TRIF–TRAF3–IKKε/TBK1–IRF3 axis to induce *Irg1* expression and ACOD1-dependent mitochondrial production of itaconate from cis-aconitate. Itaconate reshapes macrophage metabolism and inflammatory signaling through multiple mechanisms. it inhibits SDH, reducing succinate oxidation, reverse electron transport (ret), mitochondrial ROS production, and downstream HIF-1α stabilization. SDH inhibition also promotes VDAC1-dependent mtRNA release, activating the MDA5/RIG-I–MAVS–TBK1–IRF3 pathway to induce IFN-β. Itaconate activates Nrf2 through electrophilic modification of KEAP1, promoting antioxidant gene expression (e.g. HO-1 and NQO1). Itaconate also induces ATF3 and suppresses IκBζ-dependent secondary inflammatory transcription, thereby limiting IL-6 production. in parallel, itaconate targets glycolytic enzymes, including ALDOA, GAPDH, and LDHA, thereby reducing glycolytic flux. Itaconate further modulates IFNAR–JAK–STAT signaling through regulation of TYK2 and JAK1 and regulates NLRP3 inflammasome activation, GSDMD pore formation, IL-1β maturation, and pyroptosis in a context-dependent manner. Created with BioGDP.com.

##### Itaconate constrains inflammatory injury via SDH inhibition

Succinate dehydrogenase (SDH; Complex II) is a critical enzyme that links the TCA cycle to the electron transport chain. In LPS-activated macrophages, elevated SDH activity promotes succinate oxidation, thereby driving a pro-inflammatory phenotype [35]. Endogenously produced ITA inhibits SDH activity, thereby limiting succinate oxidation. Since SDH-dependent succinate oxidation drives reverse electron transport at complex I and mitochondrial ROS production, ITA-mediated SDH inhibition may suppress mitochondrial ROS generation [15,35,36]. This decrease in ROS suppresses the stabilization of hypoxia-inducible factor-1α (HIF-1α) and downregulates IL-1β transcription, ultimately limiting excessive inflammatory responses [37–39]. In infection models, a moderate inflammatory response is sufficient to control pathogens, whereas excessive inflammation results in tissue damage and impaired host defense capabilities [40,41]. For instance, during sepsis-associated inflammation, endogenously produced ITA generated through the ACOD1 pathway regulates macrophage metabolic reprogramming by inhibiting SDH activity, thereby limiting excessive inflammatory responses and contributing to organ protection [15,42]. By targeting SDH, ITA helps maintain a balance between effective pathogen clearance and protection of host tissues, highlighting its “tissue-protective” role in anti-infectious immunity.

Beyond its role in controlling inflammation, SDH inhibition by ITA also contributes to mitochondrial signaling and innate immune activation. Recent studies have shown that endogenously produced ITA-mediated inhibition of SDH elevates mitochondrial membrane potential, thereby promoting the release of mitochondrial RNA (mtRNA) into the cytoplasm through voltage-dependent anion channel 1 (VDAC1). The released mtRNA predominantly exists in a double-stranded form, which can be recognized by cytosolic RNA sensors RIG-I and MDA5. This recognition activates the MAVS-TBK1-IRF3 signaling axis, leading to theinduction of IFN-β [43]. O’Carroll S.M. et al. further demonstrated through systematic functional analyses that this effect is independent of the classical anti-inflammatory pathways of ITA, such as Nrf2 or ATF3. Instead, it is mediated by a mitochondrial stress response directly triggered by SDH inhibition, revealing a novel mechanism underlying its function as an endogenous antiviral metabolite [43]. Earlier studies also support this mechanism: Daniels B.P. et al. reported that, in neurons, *Irg1*-derived ITA restricts Zika virus replication via SDH inhibition [44], while Sohail A. et al. showed that exogenously administered ITA and its derivative 4-OI suppress viral replication and modulate host inflammatory responses during influenza infection partly through SDH inhibition [45]. These findings support the concept that ITA exerts antiviral effects through the SDH–mtRNA–IFN axis. In summary, by inhibiting SDH, ITA performs a dual function in anti-infectious immunity: limiting immunopathological damage and triggering the innate antiviral response.

##### Itaconate strengthens antioxidant defenses via Nrf2 activation and glycolytic restriction

Nrf2 is a key transcription factor regulating the antioxidant response [46]. Under resting conditions, Nrf2 binds to KEAP1 and is degraded by the proteasome [47]. ITA, through its α,β-unsaturated carboxylic acid structure, undergoes a Michael addition reaction with multiple cysteine residues (C151, C257, C288, C273, C297) on the KEAP1 protein [48]. This alkylation modification induces a conformational change in KEAP1, preventing the degradation of Nrf2 and promoting its nuclear translocation. Once in the nucleus, Nrf2 upregulates the expression of antioxidant enzymes such as HO-1 and NQO1, thereby enhancing ROS scavenging capacity [49–51]. The activation of Nrf2 is crucial for the host’s defense against infection: it protects immune cells from oxidative damage, maintains their sustained bactericidal function, and simultaneously suppresses oxidative stress-sensitive transcription factors such as NF-κB, thereby preventing uncontrolled inflammation [48]. Recent studies have shown that, in mammary epithelial cells infected with *Streptococcus uberis* (*S. uberis*), *Irg1* expression is markedly upregulated, leading to the intracellular accumulation of ITA. This accumulation promotes the clearance of excess mitochondrial reactive oxygen species through activation of the KEAP1/Nrf2 signaling pathway, thereby alleviating mitochondrial dysfunction and enhancing the expression of antioxidant enzymes such as HMOX1, NQO1, and SOD1. Furthermore, the study demonstrated that pretreatment with exogenous 4-OI effectively mitigates oxidative damage induced by *S. uberis* infection in mammary epithelial cells [52].

Enhanced glycolysis is a metabolic hallmark of inflammatory macrophages, providing energy and substrates for the synthesis of pro-inflammatory mediators [53]. ITA regulates glycolytic metabolism through covalent modification of key metabolic enzymes. Endogenous ITA has been shown to alkylate glycolytic enzymes including aldolase A (ALDOA) at C73 and C339 sites and lactate dehydrogenase A (LDHA), thereby suppressing glycolytic activity [54]. In addition, the ITA derivative 4-OI inhibits glycolysis by targeting GAPDH, contributing to its anti-inflammatory effects [55].

In host defense against infection, the inhibition of glycolysis may exert a protective effect via a dual mechanism: on the one hand, by limiting the nutrients available to pathogens, and on the other, by reducing the metabolic support required for excessive inflammation. Notably, the inhibitory effect of ITA on glycolysis acts in synergy with its Nrf2-activating effect, jointly shaping a metabolic environment conducive to pathogen clearance [42].

##### 4-OI engages additional signaling targets beyond the endogenous itaconate landscape

Although endogenous ITA primarily regulates immune responses through metabolic remodeling, including SDH inhibition, TCA cycle alteration, and moderate electrophilic modification, pharmacological 4-OI exhibits additional signaling activities due to its enhanced cellular uptake and stronger alkylation capacity. Beyond SDH inhibition, 4-OI modulates type I interferon signaling by alkylating cysteine 192 of TYK2 and cysteine 189 of JAK1, thereby disrupting TYK2/JAK1 association with IFNAR1/IFNAR2 and attenuating downstream STAT1/STAT2 activation and interferon-stimulated gene (ISG) expression [56]. This direct inhibition of the JAK-STAT pathway provides an additional layer of regulation of antiviral immunity and complements the SDH–mtRNA–IFN axis.

Furthermore, 4-OI has been shown to regulate additional inflammatory and cell-death-associated pathways through distinct post-translational mechanisms. In inflammatory signaling, 4-OI directly alkylates STING at multiple cysteine residues (Cys65, Cys71, Cys88, and Cys147), thereby suppressing STING phosphorylation and subsequently inhibiting the downstream TBK1–IRF3 axis, leading to reduced type I interferon and pro-inflammatory cytokine production [57]. In addition, 4-OI regulates ferroptotic cell death by modulating GPX4 activity. 4-OI-mediated electrophilic modification of GPX4 at Cys66 enhances its enzymatic activity, promoting lipid peroxide detoxification and preventing ROS accumulation and ferroptosis [58]. These pharmacological activities highlight that 4-OI possesses biological properties beyond those of endogenous ITA and should therefore be interpreted as a distinct therapeutic compound rather than simply an ITA surrogate [56–58].

#### Itaconate and derivatives fine-tune inflammatory signaling to optimize anti-infection responses

##### Itaconate induces ATF3 to constrain pro-inflammatory cytokines

ACOD1-derived endogenous ITA has emerged as an important immunometabolic regulator. ITA-mediated cellular stress responses can activate transcriptional programs involving ATF3, a stress-responsive transcription factor induced by endoplasmic reticulum stress pathways [59]. Once activated, ATF3 suppresses the production of pro-inflammatory cytokines such as IL-6 by inhibiting IκBζ (encoded by NF-κBζ) [60]. Through this mechanism, ITA prevents excessive amplification of immune responses during infection, thereby maintaining a balance between pathogen clearance and tissue protection. During influenza A virus infection, endogenous ITA and 4-OI exert immunomodulatory effects by suppressing excessive inflammatory signaling. Sohail et al. demonstrated that endogenous ITA and 4-OI reduce type I interferon responses, decrease pro-inflammatory cytokine and chemokine production, including CXCL10 and CCL2, and inhibit STAT1 phosphorylation and ROS production during influenza infection. In vivo, *Irg1* deficiency exacerbated pulmonary inflammation and increased mortality, whereas 4-OI administration alleviated tissue injury and improved survival [45]. Mechanistically, these anti-inflammatory effects are associated with the electrophilic activity of ITA, which activates the ATF3–IκBζ regulatory axis and subsequently restrains NF-κB-dependentinflammatory gene expression [60].

##### Itaconate modulates NLRP3 inflammasome activation in a context-dependent manner

The regulation of the NLRP3 inflammasome by ITA exhibits cell-type-dependent complexity. In classical macrophages, ITA and its derivatives inhibit NLRP3 activation: 4-OI directly modifies the C548 site of the NLRP3 protein, blocking its interaction with NEK7 and thereby inhibiting inflammasome assembly and caspase-1 activation [61]; endogenous ITA, on the other hand, modifies the C77 site of gasdermin D, thereby reducing pyroptosis and IL-1β release [62]. In influenza virus infections, ITA and 4-OI attenuate the interferon response and dampen inflammation, while simultaneously inhibiting activation of the NLRP3 inflammasome. These combined effects help restrict viral replication and mitigate pulmonary pathological damage [45]. Notably, ITA and 4-OI exhibit antiviral activity against multiple pathogens, including SARS-CoV-2, Zika virus, and influenza virus, with mechanisms closely associated with modulation of NLRP3 signaling [63]. Moreover, regulation of the NLRP3 inflammasome during *Staphylococcus aureus* (*S. aureus*) infection can significantly influence the host’s bacterial clearance capacity [64]. In the context of *M. tuberculosis* infection, the bacterial protein kinase PknG promotes degradation of NLRP3 by targeting the LUBAC complex, thereby enabling the pathogen to evade host immune surveillance [65].

However, in tissue-resident alveolar macrophages, endogenous ITA actually enhances NLRP3 activation and IL-1β production, exacerbating acute lung injury [5]. This contradiction reflects differences in the metabolic states of immune cells across distinct microenvironments, as well as biochemical distinctions between endogenous ITA and synthetic derivatives. From an anti-infectious perspective, the complexity of NLRP3 regulation suggests that the role of ITA in infectious diseases requires careful evaluation: Moderate inhibition may prevent excessive inflammation, but in specific cell types it may conversely enhance inflammation, providing an important caution for future therapeutic strategies targeting the ITA pathway.

## Mechanisms of pathogenic resistance to itaconate

Pathogens are not passive targets of ITA-mediated immunity. In response to ITA-mediated immunity, diverse bacterial species have evolved complementary strategies to evade, neutralize, or even exploit this metabolite. This section reviews three such strategies: enzymatic degradation of ITA via the conserved Ict–Ich–Ccl pathway, redirection of carbon flux toward extracellular polysaccharide synthesis and biofilm formation, and remodeling of the host immune microenvironment to favor tolerance over sterilizing immunity. Because these adaptations have evolved under the pressure of ACOD1-dependent ITA production during natural infection, the mechanistic studies discussed below primarily involve either host-derived ITA or free acid in cell-free systems.

### Enzymatic degradation of itaconate

Some intracellular pathogens have evolved dedicated mechanisms to counteract host-derived ITA, allowing them to survive under the metabolic pressure imposed by activated macrophages. ITA degradation pathways were initially identified based on enzymatic activity in several bacteria, including *Micrococcus*, *Salmonella*, and *Pseudomonas aeruginosa (P. aeruginosa*); however, the corresponding genetic determinants were not characterized until the identification of the ITA degradation cluster in *S. typhimurium* in 2014 [66]. Comparative genomic and biochemical analyses have revealed that bacterial ITA degradation systems can be broadly classified into two homologous enzyme families, which differ mainly in their CoA-transferase components and substrate activation mechanisms.

One representative pathway is the Ict–Ich–Ccl-dependent ITA degradation pathway, encoded by homologous *ict-ich-ccl* gene clusters found in diverse pathogens, including *S*. *typhimurium*, *Yersinia pestis, Brucella*, *P. aeruginosa*, *Burkholderia cepacia*, and *M. tuberculosis* [6,66–70]. In this pathway, free ITA is first activated into itaconyl-CoA by ITA-CoA transferase (Ict), followed by hydration of itaconyl-CoA into (S)-citramalyl-CoA by itaconyl-CoA hydratase (Ich), and subsequent cleavage into acetyl-CoA and pyruvate by (S)-citramalyl-CoA lyase (Ccl) (Figure 5). Although this pathway is evolutionarily conserved, bacterial species exhibit distinct CoA donor preferences and substrate activation strategies. For example, *Yersinia pestis* can utilize multiple CoA donors for ITA activation, whereas *P. aeruginosa* exhibits a more restricted preference and primarily relies on succinyl-CoA as the CoA donor [66]. A second class of ITA utilization systems has also been identified in several environmental and pathogenic bacteria. These systems differ from the classical Ict-dependent pathway and may employ alternative mechanisms for ITA uptake or metabolism. Among these alternative ITA utilization systems, *M. tuberculosis* represents a distinctive example due to its adaptation to the intracellular macrophage environment. Although macrophage-derived ITA acts as an antimicrobial metabolite during infection, *M. tuberculosis* has evolved mechanisms to exploit this host-derived compound. The Mce1 (mammalian cell entry 1) transporter complex facilitates ITA uptake into bacterial cells, providing an alternative route independent of the initial Ict-mediated CoA activation step required in the classical Ict–Ich–Ccl pathway [71]. Following uptake, ITA-derived intermediates are proposed to enter a noncanonical degradation pathway involving the bifunctional β-hydroxyacyl-CoA lyase Rv2498c, which catalyzes the cleavage of (S)-citramalyl-CoA into acetyl-CoA and pyruvate, enabling *M. tuberculosis* to utilize ITA as a carbon source. Deletion of *rv2498c* impaired bacterial growth on ITA as the sole carbon source and resulted in citramalate accumulation, confirming its role in ITA dissimilation [71]. This specialized utilization strategy highlights the metabolic flexibility of *M. tuberculosis*, allowing the pathogen to convert a host antimicrobial metabolite into a nutritional resource during infection [71].

**Figure 5.**
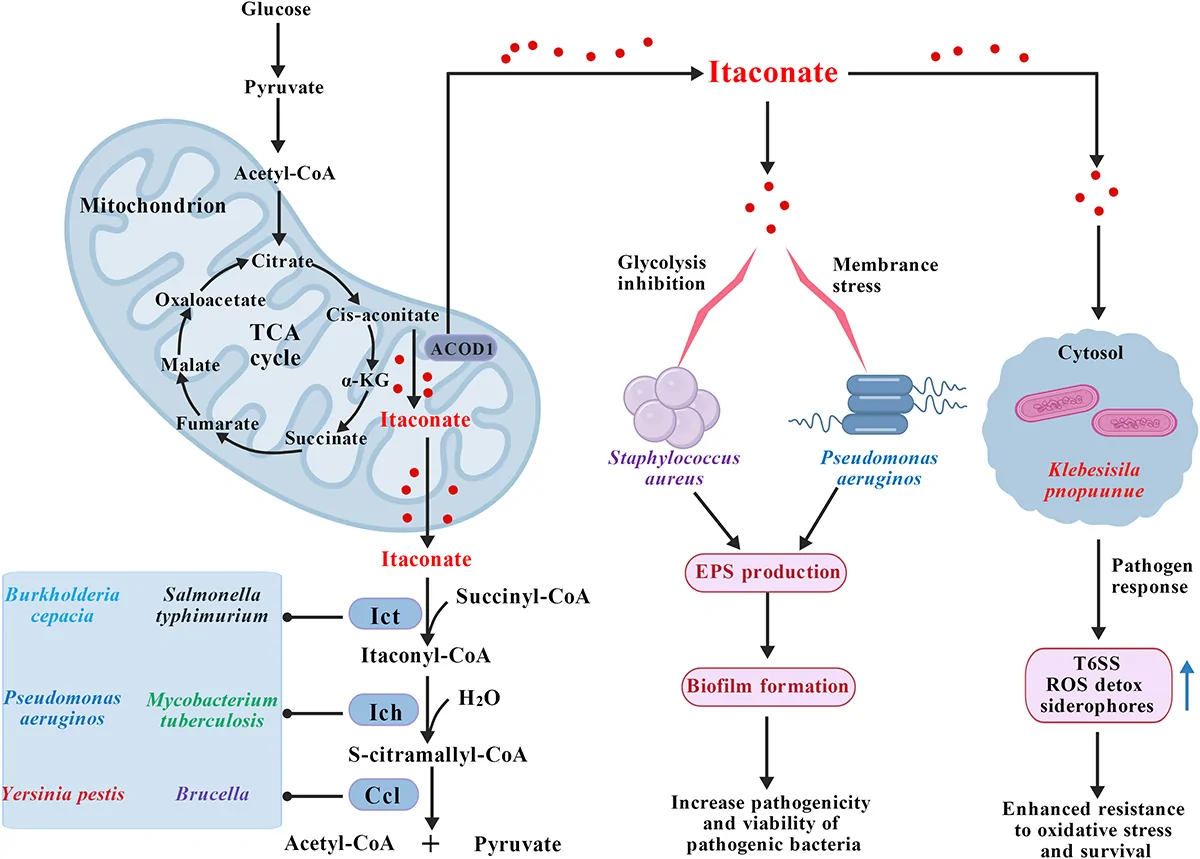
Pathogenic adaptation and resistance mechanisms to host-derived itaconate. Inflammatory activation induces mitochondrial ACOD1-dependent production of itaconate from cis-aconitate, followed by its export to exert antimicrobial pressure. in response, diverse pathogens deploy complementary adaptive strategies. Several bacteria, including *Brucella, Yersinia pestis*, *Mycobacterium tuberculosis, Pseudomonas aeruginosa, Burkholderia cepacia*, and *Salmonella typhimurium*, enzymatically degrade itaconate through a conserved three-step pathway (ict–Ich–Ccl), converting itaconate into acetyl-CoA and pyruvate to reintegrate it into central metabolism. Meanwhile, in *Staphylococcus aureus* and *Pseudomonas aeruginosa*, itaconate induces glycolytic inhibition or membrane stress, promoting extracellular polysaccharide production and biofilm formation, thereby enhancing bacterial viability and pathogenicity. in addition, *Klebsiella pneumoniae* responds by upregulating oxidative stress defense systems, siderophore production, and type VI secretion system (T6SS) components, strengthening resistance to reactive oxygen species and supporting intracellular survival. Created with BioGDP.com.

The physiological relevance of these pathways has been demonstrated through both in vitro ITA utilization and tolerance assays using free itaconic acid and infection models evaluating bacterial survival under host-derived ITA exposure. In *M. tuberculosis*, when free ITA was supplied at 10 mM as the sole carbon source, deletion of the ITA-CoA hydratase gene Rv3389c completely abolished bacterial growth and markedly impaired intracellular replication in primary macrophages, reducing bacterial burdens to approximately 20% of those observed in wild-type strains. Furthermore, Rv3389c-deficient strains exhibited significantly reduced bacterial loads in the lungs and spleens of infected mice and guinea pigs, demonstrating that ITA degradation contributes to bacterial persistence and virulence in vivo [6]. Similarly, in *P. aeruginosa*, deletion of the ITA utilization genes PA0882/PA0883 impaired bacterial growth under 4 mM free ITA conditions and reduced intracellular survival within macrophages, indicating that ITA metabolism plays an important role in bacterial adaptation and persistence [66,72]. In *S*. *typhimurium*, disruption of the ITA degradation cluster compromises bacterial replication in macrophages and attenuates virulence, highlighting the importance of this pathway in counteracting Rab32-mediated ITA-dependent host defense [32].

Interestingly, bacterial degradation of ITA does not simply eliminate an antimicrobial metabolite but may also generate additional metabolic consequences. In *M. tuberculosis*, degradation of ITA produces the intermediate itaconyl-CoA, which forms a stable biradical with the 5′-deoxyadenosyl moiety of vitamin B12-dependent methylmalonyl-CoA mutase (MCM), resulting in irreversible enzyme inactivation and impaired bacterial growth on propionate-containing substrates [73]. Therefore, bacterial ITA metabolism represents a complex adaptive strategy in which detoxification of host-derived ITA is coupled with metabolic remodeling. Although these pathways provide pathogens with a selective advantage against immunometabolic defense, the generation of reactive intermediates such as itaconyl-CoA may also impose additional physiological constraints, highlighting the evolutionary trade-offs associated with bacterial adaptation to host-derived ITA stress.

### Biofilm formation promoted by itaconate

*P. aeruginosa* and *S. aureus* can exploit host-derived ITA to enhance biofilm formation [7,68]. In *P. aeruginosa*, ITA functions primarily as an electrophilic metabolite that induces a membrane-associated stress response, thereby perturbing membrane lipid composition and intracellular redox homeostasis. This disruption compromises the membrane structural biosynthesis pathway centered on LPS. Concurrently, ITA reprograms central carbon metabolism by modulating key metabolic junctions – particularly the interface between the TCA cycle and carbohydrate metabolism – as well as associated regulatory networks. As a result, carbon flux is diverted away from the dominant LPS biosynthetic branch toward extracellular polysaccharide (EPS) synthesis pathways, including alginate, Pel, and Psl. This metabolic redistribution reduces reliance on oxidative phosphorylation while promoting the accumulation of reducing equivalents, thereby enhancing bacterial tolerance to ROS. Ultimately, increased EPS production facilitates robust biofilm formation, which was experimentally confirmed by crystal violet staining-based quantification of total biofilm biomass following ITA exposure, enabling *P. aeruginosa* to persist under oxidative stress and immune pressure and promoting the establishment of chronic infections [68]. Similarly, in *S. aureus*, ITA exerts its effects by directly inhibiting aldolase, a key enzyme in glycolysis, thereby markedly reducing carbon flux through this pathway. This inhibition blocks the cleavage of fructose-1,6-bisphosphate and leads to the accumulation of upstream intermediates such as fructose-6-phosphate. Consequently, carbon flux is redirected toward the amino sugar biosynthesis pathway, enhancing the conversion of fructose-6-phosphate to N-acetylglucosamine-6-phosphate and subsequently promoting the production of key EPS precursors, including UDP-N-acetylglucosamine. In parallel, ITA induces the upregulation of gluconeogenic pathways, ensuring a sustained supply of carbon substrates for polysaccharide biosynthesis [7]. This coordinated metabolic reprogramming ultimately drives increased EPS production and biofilm formation, thereby enhancing resistance to oxidative stress and immune-mediated clearance and facilitating the persistence of chronic infection.

### Immune microenvironment remodeling by altering itaconate production

In the context of *Klebsiella pneumoniae* (*K. pneumoniae*) ST258 pulmonary infection, the accumulation of ITA represents a host adaptive response that remodels the immune microenvironment toward a disease-tolerant state. The study by Wong Fok Lung et al. demonstrated that metabolically active *K. pneumoniae* triggers host glutaminolysis and fatty acid β-oxidation, processes that enhance mitochondrial oxidative phosphorylation and reactive oxygen species (ROS) production [74]. This metabolic stress upregulates the host enzyme ACOD1, leading to robust synthesis of ITA within macrophages and myeloid-derived suppressor cells (MDSCs). Elevated ITA exerts dual effects: it suppresses proinflammatory M1 macrophage polarization by inhibiting succinate dehydrogenase and NF-κB–mediated cytokine expression, while simultaneously stabilizing anti-inflammatory and antioxidant pathways, including the Nrf2 axis. This metabolic reprogramming favors the expansion of immunosuppressive MDSCs and M2-like macrophages, reshaping the local immune landscape to limit excessive inflammation and tissue damage [74]. However, this anti-inflammatory reconfiguration also fosters bacterial persistence by attenuating bactericidal immunity and promoting host tolerance rather than clearance. In parallel, *K. pneumoniae* organisms increase the expression of glutathione-mediated ROS detoxification (peroxidases, S-transferases, *gsiD*, *soxR*, *aphA*), siderophore production (*entS*, *fepA/D/G*, *fes*), and type VI secretion system (T6SS) gene transcription, enhancing its resistance to oxidative stress and ensuring survival within the remodeled immune microenvironment [74]. Thus, by exploiting host-derived ITA, *K. pneumoniae* promotes infection tolerance while suppressing sterilizing immunity, thereby ensuring its own persistence.

## Therapeutic potentials and challenges of itaconate in anti-infection application

The mechanistic insights reviewed above establish a conceptual foundation for the therapeutic application of ITA and its derivatives in infectious diseases. This section reviews the current preclinical evidence, organized by therapeutic mechanism rather than by pathogen or compound, so that each study is evaluated in light of the three biological outcomes defined in section 3: pathogen restriction, immune-mediated clearance, and disease tolerance. Notably, although the direct antimicrobial activities of ITA against pathogen metabolic enzymes are well documented in vitro, this mode of action has not yet been translated into a therapeutic strategy in vivo. The reasons for this gap, might be the concentration discrepancy between in vitro assays and achievable in vivo levels, are discussed in next section. Accordingly, the therapeutic studies reviewed below are predominantly host-directed, acting through enhancing host immune responses, and controlling excessive inflammation or tissue damage. We conclude with a tiered framework that maps each molecular entity to its most suitable therapeutic context. Most studies discussed here have employed synthetic derivatives, DMI and 4-OI, owing to their superior pharmacokinetic properties, a smaller subset has used exogenously administered ITA. To facilitate critical evaluation, we specify the molecular entity and dosing regimen, the infection model, underlying mechanism and treatment outcomes for each study. A summary of the key findings is provided in Table 1.

**Table 1.** The therapeutic potentials of itaconate in anti-infection disease.

Entity	Pathogen / Condition	Model	Route & Dose	Key effect	Treatment outcome	Ref
DMI	*M. tuberculosis infection*	Mouse pulmonary infection	i.p., 50 mg/kg	Promoted autophagy and phagosomal maturation; suppressed excessive inflammatory cytokines	Reduced bacterial load and lung inflammation	[75]
ITA	*P. aeruginosa* infection	Mouse pulmonary infection	Intranasal, 1 mM/20 μL	Activated OXGR1; enhanced mucociliary clearance	Promoted bacterial clearance	[76]
Endogenous ITA	*S. aureus* skin infection	Mouse skin infection	Endogenous *Irg1*-dependent production	Macrophage metabolic rewiring; enhanced antimicrobial activity	Improved local bacterial control	[77]
DMI	*S. aureus* infection	Trained immunity model	250 μM (human monocytes)	Induced epigenetic and metabolic immune reprogramming	Enhanced protection and survival	[78]
4-OI	methicillin-resistant *S. aureus* lung injury	Mouse pulmonary infection	i.p., 25 mg/kg	Activated Nrf2/ARE signaling; enhanced antioxidant defense; reduced inflammatory responses and oxidative stress	Reduced pulmonary tissue injury and improved survival	[79]
DMI	Influenza A virus infection	Mouse infection model	NR*	Suppressed excessive IFN signaling and inflammatory responses	Reduced inflammation and mortality	[45]
4-OI	Septic AKI	Mouse LPS model	i.p., 50 mg/kg	Nrf2 activation; STAT3 inhibition; reduced macrophage infiltration and inflammatory mediators	Reduced renal inflammation and injury	[80]
4-OI	Sepsis	Mouse model	NR*	STING inhibition via cysteine modification	Reduced systemic inflammation	[57]
ITA	Pulmonary fibrosis	Bleomycin mouse model	Inhalation, 0.25 mg/kg	Regulated macrophage metabolism; inhibited fibrosis	Improved lung function and pathology	[81]
4-OI	Renal fibrosis	Adenine rat model	i.v., 1, 10 mg/kg/day	Inhibited TGF-β/Smad and NF-κB; reduced oxidative stress; modulated autophagy	Improved renal function	[82]

*NR: not reported.

### Enhancing host immune responses

Beyond direct antimicrobial effects, ITA and its derivatives can enhance host-directed immune responses by reshaping macrophage function, promoting pathogen clearance, and inducing long-term immune adaptation. In *M. tuberculosis* infection, DMI has been shown to enhance host-directed antimicrobial responses by promoting autophagy activation and phagosomal maturation while limiting excessive inflammatory cytokine production. In mouse models of pulmonary mycobacterial infection, systemic administration of DMI (50 mg/kg, intraperitoneal injection) reduced bacterial burdens and attenuated inflammatory lung lesions, supporting its potential as a host-directed therapeutic strategy against mycobacterial infection [75].

Similarly, ITA can enhance bacterial clearance through host-specific pathways. In a mouse pulmonary infection model of *P. aeruginosa*, intranasal administration of ITA (1 mM in 20 μL saline) activated OXGR1 signaling and enhanced airway mucociliary clearance by promoting mucus secretion and transport, thereby facilitating bacterial elimination [76]. Notably, this extracellular host-directed clearance mechanism may bypass the intracellular ITA-degrading machinery employed by *P. aeruginosa*, highlighting the potential of ITA-based strategies to overcome pathogen counter-adaptation.

In staphylococcal infection models, endogenous ITA production has also been implicated in infection-induced immunometabolic adaptation. During *S. aureus* skin infection, dermal macrophages underwent *Irg1*-dependent metabolic rewiring accompanied by increased ITA production, which enhanced macrophage antimicrobial responses and supported local host defense. These findings demonstrate that endogenous ITA accumulation contributes to macrophage-mediated bacterial control and suggest that pharmacological augmentation of ITA pathways, including supplementation with ITA or its derivatives, may represent a potential strategy to further enhance host antimicrobial defense [77].

Beyond acute immune activation, ITA derivatives can induce durable host protection through trained immunity. DMI exposure induces long-term innate immune remodeling through transcriptional, epigenetic, and metabolic reprogramming. Human monocytes were treated with 250 μM DMI for 24 h, followed by a 6-day differentiation period and secondary stimulation with LPS (10 ng/mL) or Pam3Cys (10 μg/mL) for 24 h, resulting in enhanced inflammatory responsiveness. Consistently, DMI-treated mice showed improved survival following subsequent *S. aureus* infection, indicating that DMI-mediated trained immunity provides sustained host protection against bacterial challenge [78].

### Controlling excessive inflammation and tissue damage

In addition to enhancing antimicrobial immunity, ITA and its derivatives exhibit therapeutic potential by limiting excessive inflammatory activation and protecting host tissues from infection-associated damage. In a mouse methicillin-resistant *S. aureus* (MRSA)-induced acute lung injury model, 4-OI administration (25 mg/kg, intraperitoneal injection, administered 1 h before infection) activated the Nrf2/ARE pathway, enhanced antioxidant defenses, reduced inflammatory cytokine production, inhibited neutrophil infiltration, and decreased pulmonary pathological damage. These effects were accompanied by reduced bacterial burden and improved survival, demonstrating the protective role of 4-OI in infection-associated tissue injury [79]. In vitro studies further support the anti-inflammatory and cytoprotective effects of ITA derivatives. 4-OI treatment (62.5–250 μM, 4 h) followed by stimulation with *S. aureus*-derived lipoteichoic acid (20 μg/mL, 4 h) suppressed inflammatory responses and oxidative stress in macrophages [79]. Similarly, in a mouse influenza A virus infection model, DMI treatment reduced pulmonary inflammation and mortality by suppressing excessive interferon responses and inflammatory signaling, thereby improving host tolerance to viral infection [45].

The tissue-protective effects of ITA derivatives are also evident in systemic inflammatory disorders. In LPS-induced septic acute kidney injury (AKI), 4-OI administration (50 mg/kg, intraperitoneal injection, administered 2 h before LPS challenge) activated Nrf2 signaling, inhibited STAT3 activation, reduced macrophage infiltration and inflammatory mediators including IL-1β and IL-6, and alleviated renal tissue injury [80]. In mouse sepsis models, 4-OI further suppressed systemic inflammatory responses by inhibiting STING phosphorylation through covalent modification of STING cysteine residues, thereby reducing downstream inflammatory factor production [57].

Beyond infectious diseases, ITA-mediated immunometabolic regulation may also provide protection against chronic inflammatory tissue remodeling. In a bleomycin-induced pulmonary fibrosis model, inhaled ITA administration (0.25 mg/kg, oropharyngeal delivery, twice weekly) alleviated pulmonary fibrosis by regulating airway macrophage metabolism, maintaining oxidative phosphorylation capacity, and limiting pro-fibrotic responses, thereby improving lung pathology and function [81]. Likewise, in adenine-induced renal fibrosis models, 4-OI administration (1 or 10 mg/kg/day, tail vein injection) protected against renal injury through coordinated regulation of fibrotic signaling, oxidative stress, inflammation, and autophagy, including inhibition of TGF-β/Smad and NF-κB pathways and improvement of renal function [82]. Together, these findings highlight the potential of ITA-based interventions not only to enhance antimicrobial defense but also to improve host tolerance by preventing excessive immune-mediated tissue injury.

### A tiered therapeutic framework

Based on the preclinical evidence summarized above, we propose a tiered therapeutic framework grounded in the distinct pharmacological properties of each molecular entity. Exogenous ITA, with limited membrane permeability, may be best suited for topical or inhalational applications that engage extracellular targets suchas OXGR1 [76]. DMI, as an intracellular prodrug, serves to test whether pharmacological amplification of the endogenous ITA pathway is therapeutically beneficial. 4-OI, with its expanded target spectrum (STING, JAK-STAT, GPX4), represents a distinct strategy that pursues therapeutic value beyond the physiological ITA landscape. This framework, while consistent with the available preclinical evidence, awaits validation through head-to-head comparative studies that directly benchmark ITA, DMI, and 4-OI in identical infection models, a critical direction for future investigation.

## Conclusions and future perspectives

Over the past decade, ITA has evolved from an obscure metabolic intermediate into a pivotal hub connecting pathogen defense, immune regulation and metabolic reprogramming. Originally recognized for its direct inhibition of bacterial metabolic enzymes such as ICL and MCL, ITA is now appreciated as an important regulator of host immunity – modulating SDH activity, activating the Nrf2 antioxidant pathway, suppressing NLRP3 inflammasome assembly, and rewiring glycolytic and redox circuits. In preclinical studies, ITA and its derivatives have demonstrated therapeutic potential in infectious and infection-associated inflammatory models (Table 1), primarily by restricting pathogens, enhancing host immune defenses, and limiting excessive inflammation and tissue injury.

As research into ITA continues to advance, several important directions for future investigation are becoming increasingly apparent. These can be broadly categorized into theoretical gaps and methodological challenges. For theoretical gaps, first, the mechanism by which bacteria internalize ITA remains entirely unexplored. Identifying the bacterial transporters responsible for ITA uptake and elucidating the transmembrane transport machinery is a prerequisite for understanding how ITA exerts its direct antimicrobial effects. Second, beyond the well-characterized alkylation of cysteine residues, it remains unknown whether ITA modifies other amino acids – such as lysine or histidine – and whether such modifications contribute to its antibacterial or immunomodulatory functions. Third, structural isomers of ITA, including mesaconate and citraconate, have been detected in macrophages, yet their biological activities have received little attention. Comparative studies examining their antimicrobial and immunomodulatory properties relative to ITA could reveal complementary or context-dependent roles, thereby expanding the current immunometabolic framework. Fourth, potential crosstalk between ITA-driven modifications and other post-translational processes (e.g. succinylation, glutathionylation) represents an uncharted territory that may provide a more integrated view of how ITA fine-tunes protein function in dynamic cellular environments.

For methodological challenges, first, a critical discrepancy exists between the ITA concentrations used in most in vitro antimicrobial assays (5–100 mM) and those achieved intracellularly in activated macrophages (40 μM–8 mM). Future studies should adopt concentration ranges that more faithfully recapitulate physiological conditions to ensure that observed effects are biologically relevant [83]. Second, the majority of ITA targets identified to date have been derived from cell-free or extracellular systems; whether these same proteins are modified within infected cells or in vivo remains largely unknown. Expanding infection-relevant models, including intracellular macrophage infections and animal studies, will be essential to validate previously identified targets and to quantify the contribution of ITA under pathophysiologically meaningful settings. Third, the pleiotropic nature of ITA complicates loss-of-function approaches: direct ablation of *Irg1* not only eliminates ITA production but also perturbs broader metabolic and inflammatory networks, confounding mechanistic interpretations. Alternative strategies, such as complementation with non-metabolizable analogs or conditional expression systems, are needed to dissect its specific antimicrobial contributions from its immunomodulatory effects. Finally, commonly used chemical probes (e.g. ITalk, 4-OI derivatives) differ from endogenous ITA in both reactivity and specificity [16]. Developing next-generation probes that directly capture endogenously ITA-modified proteins, ideally under native conditions and within living cells, will be a transformative step toward a more accurate and comprehensive map of its target landscape.

## Data Availability

Data availability is not applicable to this review article as no new data were created.
